# Spatio-Temporal Scale Coded Bag-of-Words

**DOI:** 10.3390/s20216380

**Published:** 2020-11-09

**Authors:** Divina Govender, Jules-Raymond Tapamo

**Affiliations:** School of Engineering, University of KwaZulu-Natal, Durban 4041, South Africa; 215023704@stu.ukzn.ac.za

**Keywords:** action recognition, Bag-of-Words, computational efficiency, real-time systems

## Abstract

The Bag-of-Words (BoW) framework has been widely used in action recognition tasks due to its compact and efficient feature representation. Various modifications have been made to this framework to increase its classification power. This often results in an increased complexity and reduced efficiency. Inspired by the success of image-based scale coded BoW representations, we propose a spatio-temporal scale coded BoW (SC-BoW) for video-based recognition. This involves encoding extracted multi-scale information into BoW representations by partitioning spatio-temporal features into sub-groups based on the spatial scale from which they were extracted. We evaluate SC-BoW in two experimental setups. We first present a general pipeline to perform real-time action recognition with SC-BoW. Secondly, we apply SC-BoW onto the popular Dense Trajectory feature set. Results showed SC-BoW representations to successfully improve performance by 2–7% with low added computational cost. Notably, SC-BoW on Dense Trajectories outperformed more complex deep learning approaches. Thus, scale coding is a low-cost and low-level encoding scheme that increases classification power of the standard BoW without compromising efficiency.

## 1. Introduction

The aim of action recognition is to autonomously classify what is being done by observable agents in a scene. Video-based action recognition is one of the most challenging problems in computer vision. Developing robust and efficient recognition algorithms is a difficult task as the trade-off between performance and computational cost must be carefully considered.

Action recognition has various useful real-world applications. For example: autonomously finding and reporting instances of theft in public spaces, real-time surveillance, intelligent surveillance and smart shopping. Currently, state-of-the-art action recognition algorithms revolve around two-stream Convolutional Neural Networks (CNNs) [1]. However, these high performing recognition schemes often have high computational demands, making them unsuitable for real-world application. To successfully apply action recognition in real-world circumstances, algorithms that increase performance with low computational demands are valuable.

After video capturing, the first step of any action recognition task is to extract features from the given data. Various approaches use the Bag-of-Words (BoW) framework to compactly present the extracted features for classification [2,3,4,5]. The general approach of this framework involves clustering locally extracted features from training data to form a vocabulary (“bag”) of visual “words”. Extracted features are compared to this vocabulary to generate a frequency histogram. This histogram holds the count of occurrences of each visual “word” in the image/video. This histogram is the BoW representation of the image/video. The BoW is a popular approach due to its simplicity, flexibility, and computational efficiency. Thus, it has great potential for real-world action recognition problems. However, the standard framework lacks structure and discards all large-scale spatial information which reduces classification power.

Many efforts have been made to reduce the complexity of existing high performing algorithms for real-time video-based action recognition. Shi et al. [6] replaced dense sampling with random sampling, thereby reducing the number of sampled patches to process and increasing efficiency. To counteract the reduction in performance caused by the alternate sampling strategy, Shi et al. [7] increased accuracy by increasing the sampling density. This was accomplished by using a Local Part Model and performing sampling at lower spatial resolutions. Van Opdenbosch et al. [8] exploited the relationship between the generated BoW histogram and the visual vocabulary to produce a residual vector. The binary nature of this vector allows for easier and more effective compression, improving efficiency. Zhang et al. [9] replaced optical flow with Motion Vectors to increase efficiency. To boost performance, the knowledge learned with optical flow CNNs was transferred to the Motion Vectors for effective real-time action recognition. Chen et al. [10] isolated the human body from its background by person object detection. In this manner, computational resources were not wasted on processing background information.

**Figure 1:**
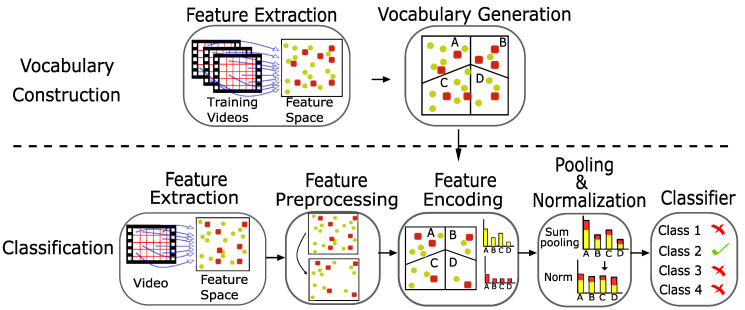
The pipeline for video-based action recognition using the Bag of Words (BoW) framework.

Conversely, many efforts have been made to increase the classification power of simple, computationally efficient algorithms, like the BoW framework, for accurate real-time applications. This was often done by combining the framework with accurate, computationally expensive classification algorithms. These include Fisher Vectors [2,11,12], CNNs [4,13] and dense sampling strategies [3,14,15]. Peng et al. [2] fused together several high-performing schemes at each step of the BoW pipeline (Figure 1) to form a hybrid scheme that maximizes performance. Although these approaches have yielded competitive results, the overall complexity is increased, resulting in higher computation resource demands. As a result, it may be difficult to achieve real-time video-based action recognition with these algorithms in real-world circumstances. For practical real-time action recognition, a notable variant is the scale coded BoW [16].

Scale coding involves encoding the spatial information of extracted patches into the final BoW representation of an image. This is a relatively simple approach yet has outperformed more complex approaches in image based action recognition [16]. Furthermore, its simplicity allows it to be incorporated into other methods, offering the same flexibility as the original BoW approach. Khan et al. [13] combined the scale coding approach with CNNs to increase performance. Thus, scale coding increases accuracy while maintaining the flexibility and computational efficiency of the original BoW framework. As a result, this algorithm has high potential for successful real-time action recognition. However, this approach has only been implemented for image-based action recognition.

Various well-performing image classification algorithms that operate in a 2-dimensional space have been extended to the third dimension of time for classification of video data. Scovanner et al. [17] extended Scale-Invariant Feature Transforms (SIFT) [18,19] to form the 3D-SIFT descriptor for classification of video data represented by the BoW approach. Other 3D extensions include extended SURF (ESURF) [20], Local Trinary Patterns (LTP) [21] and HOG3D [22]. In a similar manner, this paper aims to extend scale encoded BoW representations to the spatio-temporal domain for efficient action recognition of videos.

In this paper, we explore the potential of scale encoded BoW representations for video-based action recognition by extending the image-based scale coded BoW [16] to the spatio-temporal domain. We call this the “Spatio-Temporal Scale Coded Bag-of-Words”. We will refer to this as SC-BoW. As per the image-based approach [16], two scale coding approaches are defined: absolute and relative scale coding. Additionally, we propose two strategies for scale parameter definition: Static Scaling Parameter Definition and Dynamic Scaling Parameter Definition. To evaluate this encoding scheme, we conduct two sets of experiments on the KTH [23] and HMDB51 [24] datasets. For the first experiment, we propose a general video-based action recognition pipeline that utilizes the SC-BoW for feature representation. This pipeline is designed to operate in real-time. In the second experiment, we directly observe the performance increase SC-BoW representations can achieve by scale coding Dense Trajectory features [3].

Section 2 surveys existing work related to the study conducted. Section 3 covers the knowledge required to understand the proposed action recognition framework. Section 4 outlines the formation of SC-BoW representations. In Section 5, we present a general, real-time pipeline for action recognition with SC-BoW representations and discuss the experimental results obtained. We evaluate SC-BoW again by scale coding the Dense Trajectory feature set [3] in Section 6. Finally, Section 7 concludes the paper and outlines future work directions. The implemented code can be found online (https://github.com/divsg20/ScaleCoding).

## 2. Related Work

The BoW framework, initially developed for text categorization applications, has been adopted in visual tracking and action recognition tasks, forming the Bag of Visual Words (BoVW) framework [2,25,26]. This is alternatively referred to as the Bag of Features (BoF) framework due to the clustering of extracted features [4]. Use of this framework and its variants have dominated the research space of visual-based action recognition [2,3,4,5,13,25]. The BoW is favored due to its flexibility, simplicity, compact feature representation and computational efficiency [27]. This is useful in action recognition tasks which involve large sets of extracted features [3]. The general pipeline of this framework involves: feature extraction, vocabulary generation, feature pre-processing, feature encoding and pooling and normalization [2].

The standard BoW representation of an image or video discards all large-scale spatial information, including relative locations, scales, and orientations of extracted features [28]. This strategy justifies the compact and computationally efficient nature of the BoW framework. However, the exclusion of this information reduces the classification power of the framework. Shi et al. [11] showed that Fischer Vectors (FVs) outperform BoW representations as FVs offer a more complete representation of the dataset by including information regarding the samples’ distribution with respect to words in the vocabulary. Similarly, Loussaief et al. [29] found that CNNs outperform the BoW framework in classification tasks due to their superior ability to extract features that contain relevant information from the input data. However, FVs and CNNs are complex and high-dimensional, making them computationally expensive. To address this concern, many variants of the BoW framework were developed. The implemented modifications in BoW variants work to overcome the weaknesses of the standard BoW model. Peng et al. [2] provided a comprehensive survey, outlining the notable efforts made at each step of the BoW pipeline.

The accuracy of the BoW algorithm is dependent on the way extracted spatial and temporal information is represented, i.e., how motion features are crafted. Thus, earlier works focused on postulating powerful spatio-temporal features. Popular hand-crafted features include: Dense Trajectories [3,14,30], Space-Time Interest Points (STIPs) [31] and SIFT [18,19].

The thematic approach of BoW variants involves encoding additional information about the input data into the final BoW representation, increasing the classification power of the framework whilst leveraging the compactness and simplicity of the standard model. Laptev et al. [32] encoded spatio-temporal information into BoW representations for video-based action recognition by extending spatial pyramids used in image-based classification [33]. Li et al. [25] proposed a BoW representation that encodes contextual information of extracted features into the final representation of the image. This is referred to as the Contextual Bag-of-Words (CBOW) and was shown to outperform the standard BoW framework in visual categorization tasks. Nazir et al. [5] extended the BoW to a Bag of Expressions (BoE) by encoding neighborhood relationship information between words in the spatio-temporal domain. This achieved state-of-the-art accuracy on the KTH dataset.

Another common approach to encode relevant information into BoW representations is to combine the framework with accurate, computationally expensive video classification algorithms (e.g., deep learning algorithms). Passalis et al. [4] combined CNNs with the BoF framework to form the Convolutional Bag-of-Features (CBoF). It serves as a neural extension of the BoF framework and maintained excellent classification accuracy. This new CNN architecture uses Radial Basis Function (RBF) neurons to quantize image information for classification [4].

Current state-of-the-art video-based action recognition algorithms are dominated by deep learning methodologies: CNNs are used to effectively extract spatio-temporal information from videos [1,34] and for classification [12,35,36]. Current trends revolve around two-stream CNNs [34,37] which fuse together two CNNs for spatial and temporal information respectively. However, deep learning architectures are complex, have high computational demands and require large amounts of training data. As a result classical machine learning algorithms can be more advantageous for small data-sets such as the KTH dataset. This was confirmed by Aslan et al. [38] who achieved 95.33% on the KTH dataset using a BoW and machine learning methods. Contrarily, the deep learning algorithm by Baccouche et al. [39] achieved 94.39% on the same dataset.

## 3. Preliminary Knowledge

### 3.1. Criteria for Real-Time Action Recognition

In this section, we define the criteria to evaluate whether the proposed action recognition approach operates in real-time. Real-time processing is defined as the completion of pre-defined tasks within a set time frame [40]. The type of tasks and the length of this time frame is application dependent [41]. In action recognition applications, the system is considered to perform in real-time when the classification of the action from input data is relatively imperceptible to the user. Thus, for video-based action recognition, the criteria for real-time processing can be defined as:

(1) $$t_{p}\leq t_{c},$$

where $t_{p}$ is the processing time per frame and $t_{c}$ is the capturing time per frame.

Equation (1) can alternatively be stated as:

(2) $$f_{p}\geq f_{c},$$

where $f_{p}$ is the number of frames processed per second by the algorithm and $f_{c}$ is the number of frames captured per second.

### 3.2. Scale Invariant Bag of Words

For video-based action recognition, Peng et al. [2] defined the general pipeline for the classification algorithm using the BoW framework. This is summarized in Figure 1. A review of the well-performing existing methodologies for each step of this pipeline can be found in [2].

This section expands on the mathematical description of the standard BoW pipeline [16]. Features are extracted from a video frame via multi-scale sampling. For a given bounding box, *B*, the set of extracted features are defined as:

(3) $$F\left(B\right)=\{f_{i}^{s}|i\in \left\{1,\ldots ,N\right\},s\in \left\{1,\ldots ,M\right\}\},$$

where *i* indexes the *N* feature sites defined by the sampling grid in bounding box *B* and *s* indexes the *M* scales extracted at each feature site.

The histogram, h(B), representing a given bounding box *B* as per the BoW framework is given by:

(4) $$h\left(B\right)\propto \sum_{i=1}^{N}\sum_{s=1}^{M}c(f_{i}^{s}),$$

where *c* is some coding scheme that maps the input feature space to the representation space.

The Euclidean distance between each extracted feature $f^{s_{i}}\in F\left(B\right)$ and visual word $w_{k}$, k∈{1,...,q} in the visual vocabulary $W=\{w_{1},...,w_{q}\}$ is computed. The features are then matched to the nearest visual word (nearest neighbor assignment). The index of the visual word assigned to an extracted feature, $f_{i}^{s}$, is given by:

(5) $$\omega_{i}^{s}=\underset{k\in \{1,\ldots ,q\}}{\mathrm{argmin}}d\left(f_{i}^{s},w_{k}\right),$$

where d(a,b) is the function computing the Euclidean distance between a and b.

The coding function for the standard BoW framework is summarized by:

(6) $$c_{BOW}\left(f_{i}^{s}\right)=e\left(\omega_{i}^{s}\right),$$

where e(i) is a 1-D vector of length *q* with only one non-zero element at index *i* which is equal to 1. The index *i* corresponds to the assigned codeword for a given extracted feature $f_{i}^{s}$.

## 4. Spatio-Temporal Scale Coded Bag-of-Words (SC-BoW)

**Figure 2:**
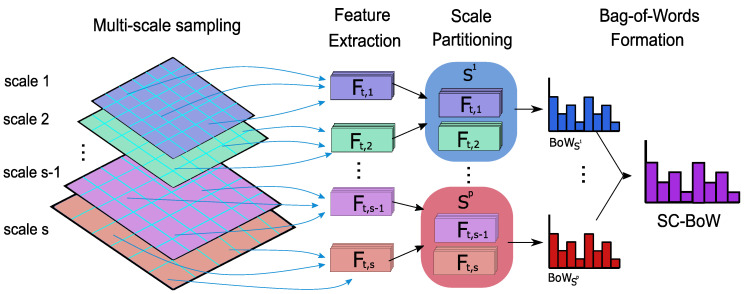
Formation of SC-BoW representations.

The general approach of scale coding (see Figure 2) involves partitioning features, sampled via multi-scale sampling, into sub-groups based on the spatial scale from which they were extracted. Thereafter, a BoW histogram is computed for each partition. The final representation is a concatenation of the histograms of each partition. For image-based action recognition, Khan et al. [16] proposed two scale coding approaches—absolute scale coding and relative scale coding—and partitioned features into three sub-groups to represent small, medium and large features.

In this section, we generalize scale coding for multiple partitions and extend both the absolute and relative encoding scheme to the spatio-temporal domain for video-based classification. For spatio-temporal extension, the following must be considered:

- Size variations of the bounding box, $B_{t}$, over each frame, *t*;
- Capturing temporal information.

### 4.1. Absolute Scale Coding

This approach encodes the scale information originally extracted from a given image patch into the final image representation. The scale of extracted features is independent of the size of the bounding box and is defined as per the original size of the image patch. Thus, the spatio-temporal extension remains largely the same as the original approach [16].

Let $S_{set}$ be the set of sampled feature scales. The scales are partitioned into *p* sub-groups as follows:

$$\begin{array}{cc} \; & S^{1}=\left\{s|s<s^{1},s\in S_{set}\right\}\\ \; & S^{2}=\left\{s|s^{1}\leq s<s^{2},s\in S_{set}\right\}\\ \; & \vdots \\ \; & S^{p-1}=\left\{s|s^{p-2}\leq s<s^{p-1},s\in S_{set}\right\}\\ \; & S^{p}=\left\{s|s^{p-1}\leq s,s\in S_{set}\right\}, \end{array}$$

where $s^{1},s^{2},...,s^{p-1}$ are the cutoff thresholds; $1<p\leq |S_{set}|$.

Let $F_{t,s}$ be the set of features extracted from an image patch on frame *t* at the spatial scale *s*, $s\in S_{set}$. The image patch is defined by the bounding box, $B_{t}$. A BoW frequency histogram is constructed for the features in each scale sub-group. The final scale coded BoW is a concatenation of each of these histograms. This is given by:

(7) $$h^{v}\propto \sum_{t=1}^{T}\sum_{s\in S^{v}}c_{BOW}\left(F_{t,s}\right),$$

where v∈{1,…,p−1,p}; *T* is the total number of frames in the video sample and $c_{BOW}$ is the BoW coding scheme defined in (6).

### 4.2. Relative Scale Coding

In this representation, extracted scale information is encoded relative to the bounding box of the object. Thus, relative scale coding is dependent on the size of the bounding box. The scale of each feature, *s*, is multiplied by a relative scaling factor, β. This factor is dependent on the dimensions of the bounding box. Since the size of the bounding box, $B_{t}$, varies from frame-to-frame, the relative scaling factor must be computed for each frame. This is defined as:

(8) $$\beta_{t}=\frac{B_{w,t}+B_{h,t}}{\bar{w}+\bar{h}},$$

where $B_{w,t}$ and $B_{w,t}$ are the width and height of the bounding box $B_{t}$ on frame *t* and $\bar{w}+\bar{h}$ is the mean width and height of all bounding boxes in the training set.

Thus, the relative extracted feature scale for each frame is defined as:

(9) $$\hat{s}=\beta_{t}s=\frac{B_{w,t}+B_{h,t}}{\bar{w}+\bar{h}}s.$$

This forms a new spatial scale set, ${\hat{S}}_{set}=\left\{{\hat{S}}_{1}\cup {\hat{S}}_{2}\cup \ldots \cup {\hat{S}}_{t}\right\}$, where ${\hat{S}}_{t}=\left\{\beta_{t}s|s\in S_{set}\right\}$. Similarly to absolute scale coding, the relative set of scale sub-groups $\hat{S^{v}},v\in \left\{1,\ldots ,p-1,p\right\}$ is partitioned as follows:

$$\begin{array}{cc} \; & \hat{S^{1}}=\left\{\hat{s}|\hat{s}<s^{1},\hat{s}\in {\hat{S}}_{set}\right\}\\ \; & S^{2}=\left\{\hat{s}|s^{1}\leq \hat{s}<s^{2},\hat{s}\in {\hat{S}}_{set}\right\}\\ \; & \vdots \\ \; & S^{p-1}=\left\{\hat{s}|s^{p-2}\leq \hat{s}<s^{p-1},\hat{s}\in {\hat{S}}_{set}\right\}\\ \; & S^{p}=\left\{\hat{s}|s^{p-1}\leq \hat{s},\hat{s}\in {\hat{S}}_{set}\right\}. \end{array}$$

The final relative scale coded representation of the image involves concatenating each BoW histogram of the scale partitions. This is given by:

(10) $$h^{v}\propto \frac{1}{|\hat{S^{v}}|}\sum_{t=1}^{T}\sum_{s\in \hat{S^{v}}}c(F_{t,s}),$$

where $|\hat{S^{v}}|$ is a normalization factor equivalent to the cardinality of set $\hat{S^{v}}$.

### 4.3. Scale Partitioning Strategies

The scale partitions are defined by the cutoff thresholds, $s^{1},s^{2},\ldots ,s^{p-1}$. In the original work [16], the cutoff thresholds are predefined parameters for a given dataset. This is a valid approach for image-based action recognition. However, in video-based action recognition, multiple scale variations can occur within a given video sample as the bounding box size changes from frame to frame in order to adapt to translation and scale variations of the target object. Thus, to accurately capture scale information, the cutoff thresholds should be dynamically set. The parameter setting scheme as per [16] will be referred to as Static Scaling Parameter Definition. The proposed dynamic setting of the cutoff thresholds will be referred to as Dynamic Scaling Parameter Definition.

For *p* scale sub-groups, the number of required cutoff threshold parameters is p−1. For dynamic definition, the cutoff thresholds, $s^{v},v\in \left\{1,\ldots ,p-1\right\}$, are set as per Algorithm 1.
**Algorithm 1:** Dynamic Scaling Parameter Definition
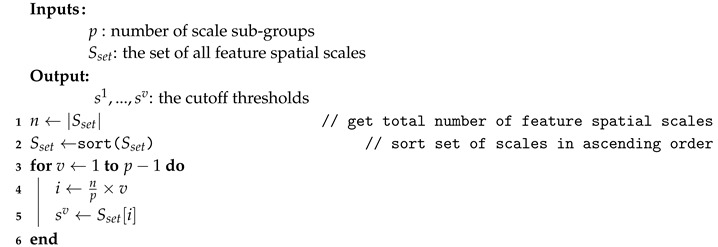


### 4.4. Scaled Spatio-Temporal Pyramids

**Figure 3:**
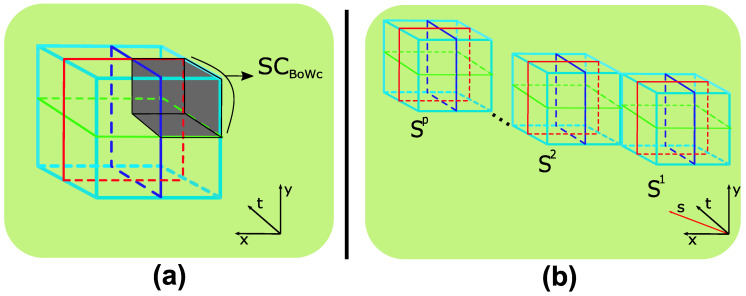
Scaled Spatio-Temporal Pyramids: (**a**) The first representation involves computing a SC-BoW for each cell. (**b**) The second representation adds scale as a 4th dimension and involves computing a standard BoW for each cell.

In action recognition tasks involving BoW representations, spatio-temporal pyramids [32] are commonly used to add additional structure and improve performance. Adding spatio-temporal structure to SC-BoW representations involves computing a SC-BoW for each spatio-temporal cell (see Figure 3a). This is defined as:

(11) $$SC_{BOW_{c}}=h^{v}\sum_{t\;inT_{p}}\sum_{s\in \hat{S^{v}}}c(F_{c,s}),$$

where $F_{c,s}$ is the set of features extracted from spatio-temporal cell *c*.

The SC-BoW for each spatio-temporal cell is pooled together and normalized to form the final scale coded spatio-temporal BoW. This effectively extends spatio-temporal pyramids to 4-dimensions as shown in Figure 3b.

## 5. Pipeline for Action Recognition with SC-BoW

**Figure 4:**
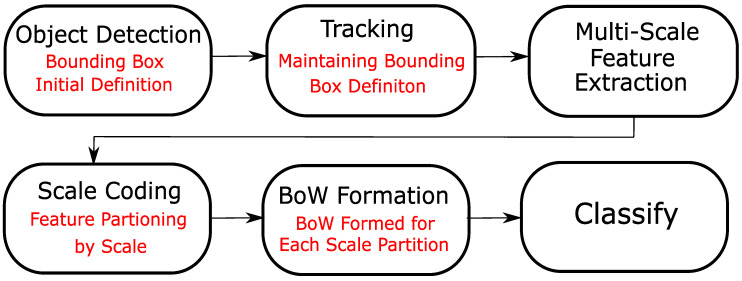
The general pipeline for video-based action recognition with scale coded BoW.

In this section, we define a general pipeline to perform video-based action recognition with absolute and relative scale coded BoW representations. This is summarized in Figure 4. For experimentation, we focus on producing a pipeline that performs action recognition in real-time. As a result, the trade-off between cost and performance is carefully evaluated when designing each step of the pipeline.

Scale coding is reliant on pre-defined bounding boxes for computation. Obtaining the bounding boxes of objects in real-time poses many additional challenges. These include: correctly and completely bounding the object and identifying which objects are important for classification.

The initial bounding box, $B_{0}$, of the target object is identified by an object detection algorithm. This bounding box is then maintained through subsequent frames via a visual tracking algorithm. For effective bounding box definition, the visual tracking algorithm must be robust to occlusion, background activities and scale variations. It must also be able to operate in real-time so that it does not hinder the overall efficiency of the action recognition algorithm.

For multi-scale feature extraction, the target object is cropped from its background. Features are extracted from this cropped image, $I_{t}$, on multiple scales. The area of $I_{t}$ is determined by the bounding box, $B_{t}$, which is defined by the visual tracking algorithm. This process is done every *R* frames. *R* represents the sampling refresh rate. *t* is the frame number.

The extracted features are represented as a SC-BoW. This is passed to a classifier for action classification.

### 5.1. Real-Time Pipeline Design

#### 5.1.1. Object Detection

For action recognition, the target object is a person. Thus, the object detection algorithm needs to not only isolate an object from its background, but also ensure that the target object is a person. Furthermore, the dimensions of the initial bounding box, $B_{0}$, must comply with a set minimum value, $dim_{min}$.

Unlike the field of action recognition where machine learning algorithms (like BoW) still remain competitive with state-of-the-art approaches, state-of-the-art object detection schemes are dominated by deep learning approaches. Since the performance of the visual tracker largely depends on the initial bounding box, deep learning-based object detectors are considered as they yield the most accurate results. As previously mentioned, deep learning schemes require large sets of labeled training data and long training times, making them difficult to implement in real-world circumstances. However, considering the widespread availability of pre-trained object detection models (https://github.com/onnx/models) with person object detection, these setbacks can be avoided.

Three common deep learning-based object detectors are Region-based CNNs (R-CNNs) and variants [42], Single Shot Detectors (SSDs) and the You Only Look Once (YOLO) detector [43]. R-CNN approaches are two-stage detectors. The first stage consists of the proposition of candidate bounding boxes. The second stage involves passing these candidate regions to a CNN for classification. As a result, R-CNN approaches are extremely accurate. However, they are extremely slow. Since the proposed action recognition algorithm aims to operate in real-time, R-CNNs are an unsuitable choice for object detection. SSDs and the YOLO detector use a one-stage detection approach and model detection as a regression problem. Although less accurate than R-CNN approaches, these are much more efficient. YOLO is the most efficient approach and is able to operate in real-time. Furthermore, it was found to achieve the best accuracy compared to other real-time detectors [43]. Thus, the YOLO object detector is used for the object detection step. Various iterations have been developed. The best performing iteration was YOLOv3 [44] which operates at 22 ms. Since the object detection algorithm is only run on the first frame to identify the initial bounding box, $B_{0}$, this is an acceptable speed.

#### 5.1.2. Visual Tracker

The tracking algorithm should be able to operate in real-time so that it does not compromise the overall efficiency of the action classification algorithm. Futhermore, the visual tracker should be able to adapt to both translation and scale variations of the target object. This is vital for our proposed action recognition algorithm; a scale encoded BoW relies on extraction of accurate scale information. The inclusion of unimportant feature information reduces the performance of the action recognition algorithm. An appropriate visual tracking model is the Discriminative Scale Space Tracker (DSST) [45].

The DSST learns two independent correlation filters to handle translation and scale variances respectively. Both models involve the training of filters for detection. To improve performance, we replace the MOSSE tracker used for the translation model with the linear Dual Correlation Filter (DCF) tracker [46] to form the DCF-DSST tracker. Implementation of the DCF-DSST tracker is summarized in Algorithm 2. The detailed implementation can be found in Appendix A. Variables for the translation and scale models are denoted with the subscripts trans and scale respectively.
**Algorithm 2:** DCF-DSST Tracker: Iteration at Frame *t* **Inputs**:
    $I_{t}$: Image patch
    $p_{t-1}$: previous frame target position
    $s_{t-1}$: previous frame target scale
    $y_{trans}$: regression target for translation model (Gaussian shaped)
    $y_{scale}$: regression target for scale model (Gaussian shaped)
 **Outputs**: 
    $p_{t}$: detected target position
    $s_{t}$: detected target scale
 **Training:**
_1_ Compute the Gaussian kernel correlation between ***x*** and itself, 
$k^{xx}$, using (A2)
_2_ Compute the DFT of the solution coefficients in the dual space, $A_{trans}$, using (A1)
 **Translation Detection:**
_3_ Construct the test sample, 
$z_{t,trans}$, from $I_{t}$ at $p_{t-1}$ and $s_{t-1}$
_4_ Compute the correlation response, 
$f(z_{t,trans})$, using (A3)
_5_ Maximize the response, 
$f(z_{t,trans})$, to find target position, $p_{t}$
 **Scale Detection:**
_6_ Construct the test sample, 
$z_{t,scale}$, from $I_{t}$ at $p_{t}$ and $s_{t-1}$
_7_ Compute the correlation response, 
$f(z_{t,scale})$, using (A10)
_8_ Maximize the response, 
$f(z_{t,scale})$, to find target scale, $s_{t}$
 **Update:**
_9_ Extract training samples 
$x_{t,trans}$ and $x_{t,scale}$ from $I_{t}$ at $p_{t}$ and $s_{t}$
_10_ Update the translation model using (A4) and (A5)
_11_ Update the scale model using (A7) and (A8) 


#### 5.1.3. Multi-Scale Sampling

For multi-scale sampling, the image patch, $I_{t}$, is cropped from the frame *t*. The area of $I_{t}$ is defined by the bounding box $B_{t}$. The number of spatial scales extracted is dependent on the dimensions of the image patch, $I_{t}$. A maximum of nine spatial scales can be extracted. As per the image-based scale coding approach [16], consecutive spatial scales are separated by a scale factor of $\sqrt{2}$. The definition of the spatial scale set is outlined in Algorithm 3. The features extracted for classification are the same as the features extracted by the visual tracker—HOG features with dimensionality reduction by Principle Component Analysis (PCA-HOG) [47]. These features can be efficiently computed and are robust to illumination and deformation. Features are sampled separately on each spatial scale $s\in S_{set}$.
**Algorithm 3:** Definition of the Spatial Scales
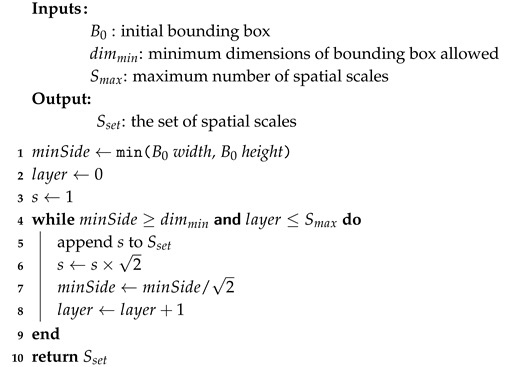


#### 5.1.4. PCA-HOG Feature Computation

Feature computation involves dividing $I_{t}$ into a grid of k×k cells and generating a HOG feature vector for each cell. Computing HOG features in this manner not only offers a more compact representation but makes the representation more robust to noise. For each cell, the gradient intensity orientation θ(x,y) and magnitude r(x,y) are computed at each pixel (x,y). In the case of color images, the θ and *r* values are taken from the color channel with the largest gradient magnitude. A bin value, b∈{0,…,l}, is assigned to each pixel based on its gradient orientation, θ(x,y). *b* is found by (12) for contrast insensitive definition or by (13) for contrast sensitive definition [47].

(12) $$B_{a}\left(x,y\right)=round\left(\frac{l\times \theta (x,y)}{\pi }\right)\;\mathrm{mod}\;l,$$

(13) $$B_{b}\left(x,y\right)=round\left(\frac{l\times \theta (x,y)}{2\pi }\right)\;\mathrm{mod}\;l,$$

where *l* is the total number of bins in the histogram.

The feature vector at each pixel, (x,y) is defined as:

(14) $$F{\left(x,y\right)}_{b}=\left\{\begin{array}{cc} r(x,y) & \;\mathrm{if}\;B(x,y)\;\mathrm{is}\;\mathrm{equal}\;\mathrm{to}\;b\\ 0 \end{array}\right.,$$

where B(x,y) denotes either $B_{a}\left(x,y\right)$ or $B_{b}\left(x,y\right)$.

The HOG feature vector, $F_{HOG}=\left[f_{1},\ldots ,f_{l}\right]$, for the k×k cell is defined as:

(15) $$f_{b}=\sum_{x=0}^{k-1}\sum_{y=0}^{k-1}F{\left(x,y\right)}_{b}.$$

The HOG feature vector used in this application is the concatenation of the contrast insensitive definition (12) with l=9 and the contrast sensitive definition (13) with l=18.

**Figure 5:**
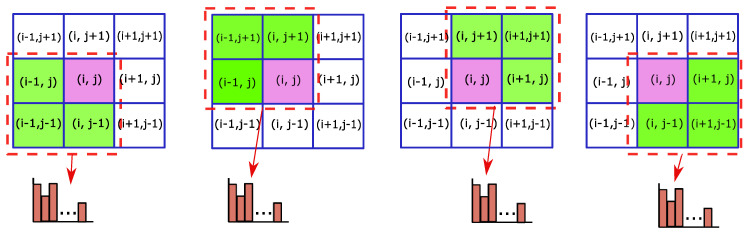
Generation of HOG features: For each block, the HOG feature vectors for the highlighted k×k cell are sum pooled and divided by the ℓ2-norm to form a normalized HOG feature histogram.

This 27-channel feature vector is block normalized by the ℓ2-norm as shown in Figure 5. This generates four normalized histograms which are then concatenated together to form a 4×(9+18)=108-channel feature vector. Thereafter, the dimensionality of the feature vector is reduced with negligible loss of information by applying PCA. This allows for more efficient computation without compromising performance. Thus, the feature map of the image patch, $I_{t}$, at the spatial scale, *s*, consists of 31-dimensional feature vectors, $F_{HOG}\left(i,j\right)$: 18 contrast sensitive orientation channels, 9 contrast insensitive orientation channels and 4 texture channels that reflect the gradient energy of the cells surrounding (i,j) [47].

#### 5.1.5. Spatio-Temporal Scale Coded BoW (SC-BoW)

The general approach for formation of SC-BoW representations was covered in Section 4. As per the image-based scale coding approach [16], we partition features into three sub-groups: small, medium and large features.

**Figure 6:**
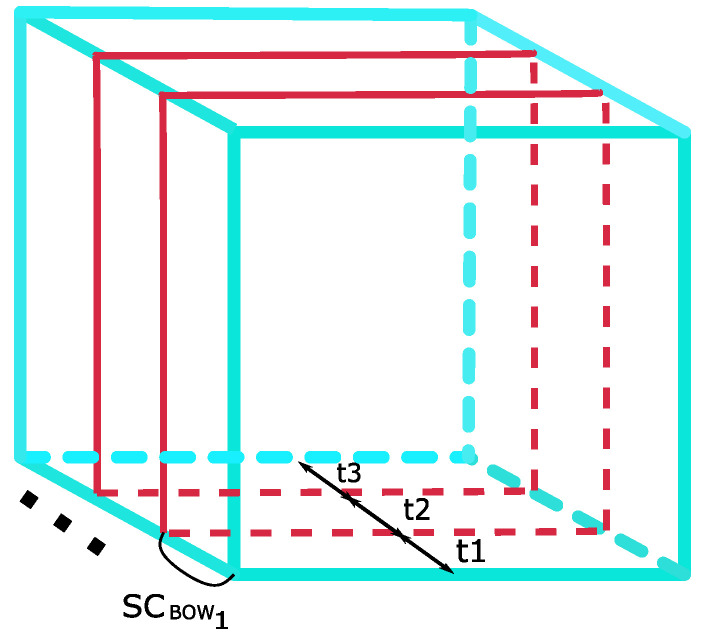
Temporal pyramid structure for SC-BoW.

The proposed algorithm excludes an optical flow algorithm since the target object is traced through the temporal domain via the visual tracking algorithm. In order to capture temporal information and add structure to the scale coded BoW, temporal pyramids are used (Figure 6). Three temporal cells are defined as:

(16) $$T_{c}=\left\{t|\left(\frac{T}{3}\times \left(c-1\right)\right)\leq t<\left(\frac{T}{3}\times c\right)\right\},$$

where *T* is the total number of frames in the video sample; t∈{1,…,T}; c∈{1,2,3}.

A scale coded BoW, $SC_{BOW_{c}}$, is constructed for each temporal cell as per (11). The scale coded BoW for each temporal cell is pooled together and normalized by the ℓ2− norm.

### 5.2. Experimental Setup

#### 5.2.1. PC Specifications

Experimentation was conducted on a PC with the following specifications: Intel Core i5 4th Gen., 1.7 GHZ, 8 GB RAM.

#### 5.2.2. Datasets

The KTH (http://www.nada.kth.se/cvap/actions/) [23] and HMDB51 (http://serre-lab.clps.brown.edu/resource/hmdb-a-large-human-motion-database/) [24] action datasets were used to evaluate performance of the proposed spatio-temporal scale encoded BoW. The classes for both datasets are balanced.

The KTH dataset contains six human action classes: walking, jogging, running, boxing, waving and clapping. Each action is performed by 25 subjects in four different environments (outdoors, outdoors with scale variation, outdoors with different clothes and indoors). This is a relatively simple dataset since the background is homogeneous and static in most sequences. Thus, the dataset serves as a minimum baseline to evaluate the ability of the proposed algorithm to perform action classification. As found in [23], samples are divided into testing and training sets based on the subjects. The testing set consists of subjects 2, 3, 5, 6, 7, 8, 9, 10, and 22 (nine subjects total) and the training set is made up of the remaining 16 subjects. The average accuracy is taken over all classes.

The HMDB51 data-set contains 51 human action classes and is collated from a variety of sources. In addition to target object translation and scale variations, this dataset includes camera motion, occlusion and background activities and thus is a much more challenging dataset. It serves as an evaluation of how robust the action recognition algorithm is to the aforementioned challenges. As per the original setup [24], each action class is organized into 70 videos for training and 30 videos for testing. Performance is measured by the average accuracy over all classes.

#### 5.2.3. Object Detection

The cvlib (https://github.com/arunponnusamy/cvlib) implementation is used. The YOLOv3 model used was trained on the COCO dataset (https://cocodataset.org/#home) and is able to detect 80 objects including a person.

The minimum required dimensions of the initial bounding box, $B_{0}$, is set to $dim_{min}=24$ pixels. A 24×24 bounding box ensures at least one feature map containing 2 PCA-HOG feature vectors can be extracted via multi-scale sampling.

#### 5.2.4. Visual Tracker

The set of parameter values for implementation of the DCF-DSST tracker is summarized in Table 1. The parameter values were set as per the original papers [45,46].

#### 5.2.5. Bag of Words

PCA-HOG (https://github.com/uoip/KCFpy/blob/master/fhog.py) features are efficiently extracted from training samples (as covered in Section 5.1.3) using a 4×4 cell grid. K-means clustering is used to cluster a subset of 100,000 randomly selected training features to construct a BoW vocabulary of 4000 words. The scale coded BoW is formed from the extracted features as outlined in Section 5.1.5. Both the absolute and relative scale coding schemes are evaluated. Furthermore, the effects of the proposed scale partitioning strategies (static and dynamic scaling parameter definition) are evaluated on the KTH dataset in order to determine which strategy yields the best results. For static parameter definition, the cutoff thresholds are set as: $s^{s}=\sqrt{2}$, $s^{l}=2$. To lower computation time, the scale coding process is parallelized; each scale sub-group ($S^{s},S^{m}$ and $S^{l}$) is computed on individual threads.

#### 5.2.6. Classification

Similarly to [16], a one-vs-rest SVM is used for multi-class classification. Three SVM kernels were considered:

1. A linear kernel [48] defined as (1)
  (17) $$K\left(x_{i},x_{j}\right)=x_{i}•x_{j}.$$
2. A $\chi^{2}$ kernel [48] defined as (2)
  (18) $$K\left(x_{i},x_{j}\right)=exp\left(-\frac{1}{A}D\left(x_{i},x_{j}\right)\right),$$
  where $D(x_{i},x_{j})$ is the $\chi^{2}$ distance between each training video $x_{i}$ and $x_{j}$. *A* is a scaling parameter.
3. An rbf kernel [49] defined as (3)
  (19) $$K\left(x_{i},x_{j}\right)=exp\left(-\gamma {∥x_{i}-x_{j}∥}^{2}\right).$$
  the kernel type, *A* and γ values are hyper-parameters that are tuned using 5-fold cross validation.

### 5.3. Results and Discussion

#### 5.3.1. Scale Partitioning Strategies

The effects of each scale partitioning strategy can be observed in Table 2. Dynamically defining the cutoff thresholds, $s^{s}$ and $s^{l}$, results in better performance compared to the static strategy. Notably, the improvement in accuracy was much higher for the relative scale encoding scheme (+3.24%) compared to the absolute scale encoding scheme (+0.47%). This was expected as the set of extracted scales for relative scale encoding, ${\hat{S}}_{set}$, is dynamically created since it is dependent on the size of the bounding box, $B_{t}$, in each frame. Contrarily, the set of extracted scales for absolute scale encoding, $S_{set}$, is created on the first frame since it is only dependent on the initial bounding box, $B_{0}$. Thus, it is more difficult to predict the cutoff thresholds that will partition extracted scales into scale sub-sets of equal size for the relative scale encoding scheme. This results in a less accurate representation of extracted scale information, lowering accuracy. It can be concluded that for video-based scale coding, dynamic definition of the cutoff thresholds ensures that extracted scale information is more accurately represented and thus results in a better performance.

#### 5.3.2. Scale Coding Schemes

Observing the results achieved on the KTH dataset (Table 2), it can be seen that the relative scale coding scheme (64.81%) achieves a slightly higher accuracy than the absolute scale coding scheme (63.89%). The same outcome was observed in the image-based scale coded BoW [16]; relative scale coding outperformed the absolute scheme by 0.7%. This may be due to the fact that the relative encoding scheme accounts for the size of the bounding box, thus encodes more information into the final representation compared to the absolute coding scheme; this results in a higher accuracy. However, for the HMDB51 dataset, the opposite is observed (see Table 3). The absolute coding scheme (27.8%) outperforms the relative coding scheme (21.24%) by a relatively large margin of 6.56%. This may be due to the the visual tracking algorithm inaccurately defining the bounding box, $B_{t}$, in each frame *t*, as the HMDB51 dataset contains occlusion, camera motion and background activities. Capturing unimportant information can reduce performance and wastes resources. Since the relative coding scheme is heavily dependent on the bounding box, the errors of the visual tracker have a greater negative impact on the relative coding scheme compared to the absolute coding scheme.

#### 5.3.3. Computational Cost

The time taken to complete each task of the proposed scale coded BoW was computed for 25 randomly selected video samples in each dataset. These values were averaged and are presented in Table 4.

As per Equation (2), the proposed pipeline operates in real-time. Although by definition, the KTH dataset does not operate in real-time since the processing frequency ($f_{p}$) is slightly less than the capturing frequency ($f_{c}$), the difference between these values is small. Thus, processing is perceived to be in real-time. Furthermore, the hardware of the PC on which experiments were conducted is outdated. Thus, the proposed action recognition pipeline will easily operate in real-time on state-of-the-art PCs.

On average, formation of the SC-BoW for a vocabulary size of 4000 words operates at 78.92 fps. This is relatively efficient (given the outdated hardware on which experiments were conducted) and is done in real-time for the proposed pipeline. The computation time of SC-BoW representations can be improved by computing the BoW using the algorithm presented in [50].

#### 5.3.4. Comparison to Existing Methods

Observing the comparisons in Table 5, it can be seen that the proposed method is outperformed by most cited existing methods. However, SC-BoW representations show an improved accuracy on the HOG feature set compared to more complex algorithms. The proposed algorithm outperforms the HOG/HOF feature-based algorithm proposed by Kuehne et al. [24] by 4.6% and the Local Part Model [6] HOG feature set by 6.78%.

#### 5.3.5. Setbacks

A large setback of the proposed pipeline is its dependence on the the performance of the visual tracker. If the visual tracker fails, large amounts of unimportant information will be captured, reducing the efficiency and performance of the algorithm. Thus, it would be beneficial to reduce the reliance of this algorithm on the visual tracker by either:

- Removing the need for a visual tracker by using alternative methods to define the target object through subsequent frames;
- Removing dependence of the relative coding scheme on the definition of a bounding box by using alternative cues to compute relative scale (e.g., depth information).

## 6. Scale Coding Dense Trajectories

The experimental results obtained in Section 5 showed SC-BoW representations to outperform more complex approaches on the HOG feature set. This highlighted SC-BoW representations as a potential low-cost solution to increase performance. In order to solidify this claim and directly observe the performance increase SC-BoW representations can achieve, we apply scale coding to the popular Dense Trajectory feature set [3].

### 6.1. Experimental Setup

#### 6.1.1. PC Specifications and Datasets

The PC specifications are the same as stated in Section 5.2. The code was run on a single CPU core. To evaluate performance of the proposed spatio-temporal scale encoded BoW, the KTH [23] and HMDB51 [24] datasets are used. The setup is the same as stated in Section 5.2.2 except a reduced HMDB51 dataset is used for evaluation as opposed to the full version. The reduced HMDB51 dataset consists of 11 randomly selected action classes: brush hair, cartwheel, catch, chew, clap, climb stairs, smile, talk, throw, turn, wave. For the reduced HMDB51 dataset, the training set consists of 70 videos and the testing set consists of 30 videos. Thus, the classes for both datasets are balanced.

#### 6.1.2. Dense Trajectories

Dense Trajectories involve densely sampling points in the spatial domain and tracking those points across the temporal domain via a dense optical flow algorithm to form a trajectory. Thereafter, five descriptors are extracted: trajectory shape, HOG, HOF and Motion Boundary Histograms in the horizontal (MBHx) and vertical (MBHy) planes [3].

Points are densely sampled on each spatial scale using a sampling step-size of W=5. There are a maximum of eight spatial scales; the number of spatial scales is dependent on the resolution of the video. Spatial scales are separated by a factor of $1/\sqrt{2}$ [3]. Sampled points in homogeneous image areas can not be tracked and are therefore removed based on the criterion presented by [51]. The threshold, *T*, is defined as:

(20) $$T=0.001\times \max_{i\in I}\min\left(\lambda_{i}^{1},\lambda_{i}^{2}\right),$$

where $\left(\lambda_{i}^{1},\lambda_{i}^{2}\right)$ are the eigenvalues of the $i^{th}$ sampled point in the image I. Wang et al. [3] set the scalar value in (20) to 0.001 since it presented a good compromise between saliency and density.

Points are re-sampled and compared to the threshold *T* every R frames. R is the refresh/frame sub-sampling rate. We set the refresh rate as per Govender et al. [52]: a refresh rate R=1 for the KTH dataset and R=6 for the reduced HMDB51 dataset yields the best performance.

To form trajectories, sampled points are tracked separately on each spatial scale via a dense optical flow algorithm [53]. The trajectories are defined as:

(21) $$P_{t+1}=\left(x_{t+1},y_{t+1}\right)=\left(x_{t},y_{t}\right)+\left(M*\omega_{t}\right){|}_{\left(x_{t},y_{t}\right)},$$

where $P_{t}$ is a point in frame $I_{t}$. $\omega_{t}$ is the dense optical flow field for each frame $I_{t}$. This is found with respect to the next frame $I_{t+1}$. *M* is the 3×3 median filter kernel applied to the optical flow field.

The same optical flow implementation (https://docs.opencv.org/3.4/d4/dee/tutorial_optical_flow.html) as [3] was used. From tracked points, five descriptors are extracted: HOG, HOF, MBH in the x (MBHx) and y (MBHy) planes and the trajectory shape, TS, defined as:

(22) $$TS=\frac{\Delta P_{t},\ldots ,\Delta P_{t+L+1}}{\sum_{j=t}^{t+L-1}||\Delta P_{j}\left|\right|},$$

where *L* is the length of the trajectory. This is set to 15 frames as per [3].

#### 6.1.3. SC-BoW Representation

Following [3], k-means clustering is used to cluster a subset of 100,000 randomly selected training features. A vocabulary of 4000 words is separately constructed for each descriptor.

The generated features are partitioned into scale sub-groups as outlined in Section 4. The original Dense Trajectory algorithm [3] does not define bounding boxes, thus only the absolute scale coding scheme is applied (see Section 4.1). The scale cutoff thresholds are dynamically set.

**Figure 7:**
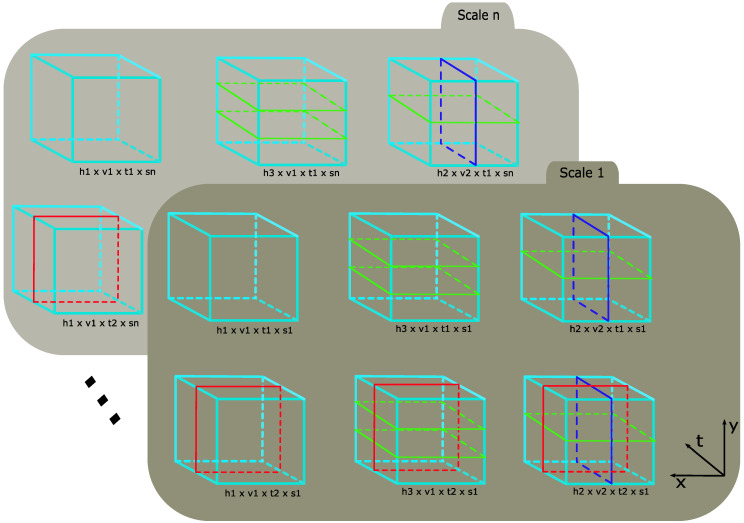
Scaled Spatio-Temporal Pyramids to add structure to SC-BoW representations.

In the original work [3], structure is added to BoW representations using six spatio-temporal pyramids. We extend the pyramid structure to hold scale information (see Figure 7). A BoW is constructed for each scaled spatio-temporal cell. Thereafter, a global representation of the pyramid is found by summing the BoW histogram for each cell and normalizing by the Root SIFT Norm [54], defined as:

(23) $$p_{k}=\sqrt{\frac{p_{k}}{\sum_{k=1}^{q}\left|p_{k}\right|}}.$$

Each pyramid-descriptor pair forms a separate channel; there are 30 channels in total (6 pyramids × 5 descriptors). A multi-class, multi-channel non-linear SVM with an RBF-$\chi^{2}$ kernel [23] is used for classification. For multi-class classification, a one-against-rest approach is used. For multiple channels, the kernel is defined as [48]:

(24) $$K\left(x_{i},x_{j}\right)=exp(-\sum_{c}\frac{1}{A_{c}}D\left(x_{i}^{c},x_{j}^{c}\right),$$

where $D(x_{i}^{c},x_{j}^{c})$ is the $\chi^{2}$ distance between each training video $x_{i}$ and $x_{j}$ in each channel *c*. $A_{c}$ is the average of the $\chi^{2}$ distances between training samples in channel *c*.

### 6.2. Results and Discussion

#### 6.2.1. Performance Analysis

The time taken to obtain SC-BoW representations from extracted dense trajectory features was recorded for 25 randomly selected video samples from the KTH dataset. These values were averaged and are presented in Table 6. The average number of extracted features clustered for each video sample was 6000. SC-BoW representations were formed with 3 partitions.

SC-BoW representations were found to improve performance on the KTH by 2.76% and on the reduced HMDB51 dataset by 3.64% (see Table 7). The increase in performance was accompanied by a 5.59s increase in computational time (Table 6). For the standard Dense Trajectory algorithm, it was experimentally found that BoW formation accounts for 2% of total computation time (9013s). Thus, incorporation of SC-BoW increases computation time by 0.06%. This is a negligible increase in most cases. Additionally, the added computation time can be further reduced by parallelizing the code. It can be concluded that SC-BoW representations increase accuracy with negligible added computational cost.

**Figure 8:**
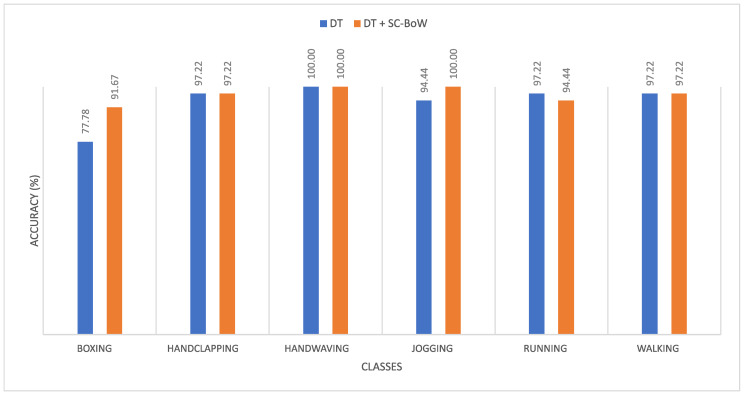
Plot comparing the class accuracies obtained on the KTH dataset for dense trajectories and scale coded dense trajectories.

**Figure 9:**
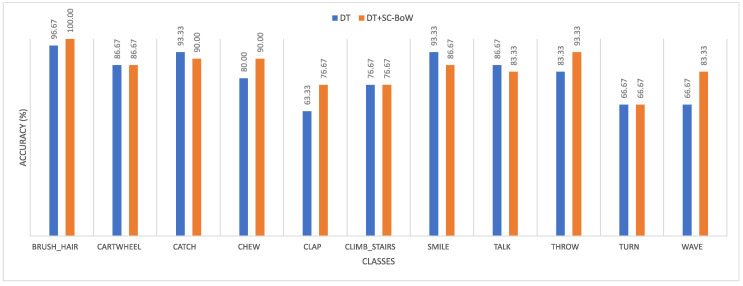
Plot comparing the class accuracies obtained on the reduced HMDB51 dataset for dense trajectories and scale coded dense trajectories.

The accuracy achieved in each class for scale coded and non-scale coded dense trajectories was recorded for both datasets. This is summarized in Figure 8 (KTH dataset) and Figure 9 (reduced HMDB51 dataset).

#### 6.2.2. Number of Scale Partitions

**Figure 10:**
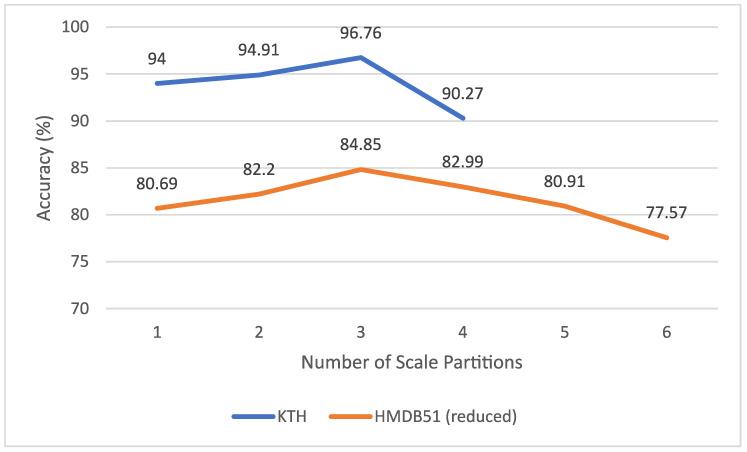
The effect of number of scale partitions on accuracy for the KTH and HMDB51 (reduced) datasets.

We observed the achieved accuracy for p∈{1,…,n} partitions. p=1 corresponds to the original algorithm with no scale coding. *n* is the maximum spatial scale and is dependent on the resolution of the video sample. For the KTH dataset, n=4. For the reduced HMDB51 dataset, n=6. Observing the results in Figure 10, there exists an optimum number of scale partitions. For both the KTH and reduced HMDB51 dataset, this was found to be p=3. Similarly, Khan et al. [13] had found a negligible gain in performance beyond 3 scale partitions for image-based action recognition. It can be concluded that forming SC-BoW representations with p=3 partitions will achieve optimal performance in most cases.

#### 6.2.3. Comparison To Existing Methods

Observing the values in Table 8, applying SC-BoW to Dense Trajectory features results in performance close to state-of-the-art. Furthermore, scale coded dense trajectories outperformed complex deep learning approaches that use the popular two-stream CNN network architecture [55]. This highlights that deep learning approaches do not always outperform machine learning approaches.

## 7. Conclusions

We encoded scale information into BoW representations for video-based action recognition to form the “Spatio-Temporal Scale Coded Bag-of-Words” (SC-BoW). The inclusion of extracted scale information into BoW representations better describes the data and, as a result, improved performance compared to the standard BoW framework. The improved performance was achieved with a low added computational cost. Additionally, SC-BoW representations were found to improve performance above more complex approaches, including deep learning approaches. Since SC-BoW is a relatively low-level encoding scheme, it can be easily incorporated into any existing algorithm that uses BoW representations for a low-cost performance boost. Thus, SC-BoW representations are a promising avenue for real-world action recognition problems. Future work includes applying SC-BoW representations to deep feature sets.

## Figures and Tables

**Table 1:** Parameters for the DCF-DSST Visual Tracker.

Parameter	Description	Value
**Parameters for Translation Model (DCF) as per** [46]		
$\lambda_{trans}$	Regularization parameter for translation model	0.0001
$\eta_{trans}$	Learning rate for translation model	0.02
**Parameters for Scale Model as per** [45]		
$\lambda_{scale}$	Regularization parameter for scale model	0.01
$\eta_{scale}$	Learning rate for translation model	0.025
*S*	Number of scales	33
sf	Scale Factor	1.02

**Table 2:** Performance of the Spatio-Temporal Scale Coded BoW on the KTH dataset.

Scale Coding	Parameter Definition	Hyper-Parameters	Accuracy (%)
Absolute	Static	kernel = ‘linear’	63.42
	Dynamic	kernel = ‘linear’	63.89
Relative	Static	kernel = ‘linear’	61.57
	Dynamic	kernel = ‘linear’	**64.81**

**Table 3:** Performance of the Spatio-Temporal Scale Coded BoW on the HMDB51 dataset with Dynamic Scale Parameter Definition.

Scale Coding Scheme	Hyper-Parameters	Accuracy (%)
Absolute	kernel = ‘rbf’, γ=0.01	**27.78**
Relative	kernel = ‘rbf’, γ=0.01	21.24

**Table 4:** The Computational Cost of Each Task in the Spatio-Temporal Scale Coded BoW for the KTH and HMDB51 Datasets.

Task	KTH	HMDB51
Object Detection	1.01 s	1.81 s
Feature Extraction	7.15 s	7.73 s
Scale Coding	5.44 s	4.35 s
Total Processing Time $t_{p}$	13.60 s	13.89 s
Average Number of frames per video	335.95	417.95
Processing Frequency $f_{p}$	24.70 fps	30.09 fps
Capturing Frequency $f_{c}$	25 fps [23]	30 fps [24]

**Table 5:** Comparison to Existing Action Recognition Methods for HMDB51.

Method	Accuracy (%)
HMDB51 [24] (Combined)	23.18
HOGHOF	20.44
HOG	15.47
HOF	22.48
Local Part Model [6] (Combined)	47.6 *
HOG	21.0
HOF	33.5
HOG3D	34.7
MBH	43.0
Motion Vector CNNs [9]	55.3 *
Scale Coded BoW	27.78

* Algorithms that operate in real-time.

**Table 6:** Computational Analysis of SC-BoW given a 4k-word vocabulary and  6000 extracted features.

Task	Computation Time (s)	Computation Frequency (fps)
BoW Formation with Scale Coding	192.24	5.22
BoW Formation without Scale Coding	186.65	6.10
Added Cost	**5.59**	**0.88**

**Table 7:** Comparing Performance of Dense Trajectories with and without scale coding for the KTH and reduced HMDB51 datasets.

Description	KTH	HMDB51 (Reduced)
DT (%)	94.0	81.21
DT + SC-BoW (%)	96.76	84.85
Net Performance Change (%)	**+2.76**	**+3.64**

**Table 8:** Comparison to Existing BoW Action Recognition Methods for the KTH dataset.

Method	Accuracy (%)
Sequential Deep Learning [39]	94.38
Spatio-temporal CNN [55]	95.86 ± 0.3
Local Part Model [6]	93.0 *
BoE [5]	99.51
DT + SC-BoW	96.76

* Algorithms that operate in real-time.

## Appendix A. DCF-DSST Visual Tracker

### Appendix A.1. Translation Model

DCF is based on the multi-channel extension of the standard Kernalized Correlation Filter (KCF) tracker [46]. This involves mapping the inputs, $x_{i}$, of a linear problem to a non-linear feature space (known as the dual space) by some mapping function ϕ(x). This is an attractive approach as the optimization problem remains linear, allowing for simple evaluation. The solution in the dual space is efficiently computed using:

(A1) $$A=\frac{Y}{K^{xx}+\lambda },$$

where Discrete Fourier Transforms (DFT) are indicated by capitalization (*A* is the DFT of α, $K^{xx}$ is the DFT of $k^{xx}$, Y is the DFT of y). α is the solution in the mapped feature space. $k^{xx}$ is the kernel correlation of x with itself. y is a vector with the elements $y_{i}$.

Using a linear kernel, $\kappa \left(x,x^{\prime }\right)=x^{T}x^{\prime }$, the kernel correlation is defined as:

(A2) $$k^{xx^{\prime }}=F^{-1}\left(\sum_{c}{\bar{X}}_{c}⊙X_{c}^{\prime }\right),$$

where the input vector $x=[x_{1},\ldots ,x_{c},\ldots ,x_{C}]$ concatenates the vectors of each channel, $x_{c}$. c∈{1,2,…,C}; *C* is the total number of channels. The complex conjugate form of *X* is indicated by a bar, i.e., $\bar{X}$.

The first frame is trained with the initial position of the target, $p_{0}$. Subsequent frames are trained on the detected position of the target, $p_{t}$. The matching scores of all cyclic shifted versions of some candidate M×N image patch, z, is computed and $p_{t}$ is set as the position that returns the maximum matching score i.e., the maximum of the response f(z). The matching score function is given by:

(A3) $$f\left(z\right)=F^{-1}\left(\hat{A}⊙K^{\hat{x}z}\right),$$

where $\hat{A}$ are the learned coefficients. $\hat{x}$ is the learned object appearance model. $K^{\hat{x}z}$ is the DFT kernel correlation between elements of $\hat{x}$ and z.

The learned coefficients and object appearance model are updated every frame by (A4) and (A5) respectively.

(A4) $$\hat{A_{t}}=\eta A_{t}+\left(1-\eta \right){\hat{A}}_{t-1},$$

(A5) $$\hat{X_{t}}=\eta X_{t}+\left(1-\eta \right){\hat{X}}_{t-1},$$

where η is the fixed learning rate. This is typically 0.02.

### Appendix A.2. Scale Model

As per [45], the scale filter for channel *c*, is defined as

(A6) $$H^{c}=\frac{\bar{Y}X_{c}}{\sum_{k=1}^{C}\bar{X_{k}}X_{k}+\lambda },$$

where *Y* is the DFT of the desired response of the filter. This is set to be 1-dimensional Gaussian response. λ is the regularization parameter of the scale filter.

To find the optimal filter, the numerator and denominator parts of Equation (A6) are trained separately. $U^{c}$ is the learned numerator for the $c^{th}$ channel. *D* is the learned denominator. The filter is updated every frame *t* using the following equations.

(A7) $$U_{t}^{c}=\left(1-\eta \right)U_{t-1}^{c}+\eta \bar{Y}X_{c},$$

(A8) $$D_{t}=\left(1-\eta \right)D_{t-1}+\eta \sum_{k=1}^{C}\bar{X_{k}}X_{k}+\lambda ,$$

where η is the learning rate parameter. $X_{c}$ is the DFT of the vector in the $c^{th}$ channel of the training sample, $x_{t}$.

The training sample, $x_{t}=\left[x_{t}^{1},\ldots ,x_{t}^{c},\ldots ,x_{t}^{C}\right]$, is constructed using the features extracted at various scales from the image patch $I_{t}$ at frame *t*. The size of the scaled image patches, $I_{t,n}$, is defined as

(A9) $${sf}^{n}M\times {sf}^{n}N,$$

where sf is the scale factor. M×N is the size of the image patch, $I_{t}$. $n\in \{-\frac{S-1}{2},\ldots ,\frac{S-1}{2}\}$. *S* is the size of the scale filter.

To populate the scale training sample set, $x_{t}$, the value $x_{t}\left(n\right)$ is the the *C*-dimensional feature vector extracted from the image patch, $I_{t,n}$ at the scale level *n*. Thus the size of $x_{t}$ is C×S.

The correlation scores for frame *t*, $f(z_{t})$, are computed using the below equation.

(A10) $$f\left(z_{t}\right)=F^{-1}\left(\frac{\sum_{c=1}^{C}{\bar{U}}_{t-1}^{c}Z_{t}^{c}}{D_{t-1}+\lambda }\right),$$

where $z_{t}^{c}$ is the vector in the $c^{th}$ channel of the test sample, $z_{t}=\left[z_{t}^{1},\ldots ,z_{t}^{c},...,z_{t}^{C}\right]$, constructed in frame *t*. This is constructed in the same manner as the training sample, $x_{t}$.

By maximizing $f(z_{t})$, the target scale is found. Thus, the bounding box for the frame *t*, $B_{t}$, is centered at $p_{t}$ and is of the size ${sf}^{s_{t}}M\times {sf}^{s_{t}}N$. $s_{t}$ is the target scale.

Following [45], PCA-HOG [47] features are extracted from the image patch, $I_{t}$ for sample construction. As detailed in [56], the extracted features are always multiplied by a Hanning Window defined by:

(A11) $$Hann\left(n\right)=0.5\left(1-\cos(\frac{2\pi n}{S-1})\right).$$
